# Immunogenicity, efficacy, and safety of SARS-CoV-2 vaccine dose fractionation: a systematic review and meta-analysis

**DOI:** 10.1186/s12916-022-02600-0

**Published:** 2022-10-25

**Authors:** Bingyi Yang, Xiaotong Huang, Huizhi Gao, Nancy H. Leung, Tim K. Tsang, Benjamin J. Cowling

**Affiliations:** 1 WHO Collaborating Centre for Infectious Disease Epidemiology and Control, School of Public Health, Li Ka Shing Faculty of Medicine, The University of Hong Kong, Hong Kong, China; 2 Laboratory of Data Discovery for Health Limited, Hong Kong Science and Technology Park, New Territories, Hong Kong, China

**Keywords:** COVID-19, Vaccine, Dose fractionation, Systematic review

## Abstract

**Background:**

Dose fractionation of a coronavirus disease 2019 (COVID-19) vaccine could effectively accelerate global vaccine coverage, while supporting evidence of efficacy, immunogenicity, and safety are unavailable, especially with emerging variants.

**Methods:**

We systematically reviewed clinical trials that reported dose-finding results and estimated the dose-response relationship of neutralizing antibodies (nAbs) of COVID-19 vaccines using a generalized additive model. We predicted the vaccine efficacy against both ancestral and variants, using previously reported correlates of protection and cross-reactivity. We also reviewed and compared seroconversion to nAbs, T cell responses, and safety profiles between fractional and standard dose groups.

**Results:**

We found that dose fractionation of mRNA and protein subunit vaccines could induce SARS-CoV-2-specific nAbs and T cells that confer a reasonable level of protection (i.e., vaccine efficacy > 50%) against ancestral strains and variants up to Omicron. Safety profiles of fractional doses were non-inferior to the standard dose.

**Conclusions:**

Dose fractionation of mRNA and protein subunit vaccines may be safe and effective, which would also vary depending on the characteristics of emerging variants and updated vaccine formulations.

**Supplementary Information:**

The online version contains supplementary material available at 10.1186/s12916-022-02600-0.

## Background

Three years into the pandemic, coronavirus disease 2019 (COVID-19) continues to threaten global health with emerging variants. While vaccinations are effective in preventing hospitalizations and deaths [1, 2], there has been unequal distribution of vaccinations across the globe. Despite that the current vaccine supply would cover most of the global population, a portion of the supply were prioritized for the fourth or fifth dose in high-income countries, while only 15.7% of people in lower-income countries had received at least one vaccine dose as of 16 May 2022 [3]. Dose fractionation of vaccines has been previously recommended to ease global supply shortage and accelerate vaccine coverage in low-income countries, where a larger proportion of the population could have access to vaccination while each individual would receive a lower vaccine dose [4, 5]. However, uncertainties and concerns about the vaccine efficacy using fractional doses against severe acute respiratory syndrome coronavirus 2 (SARS-CoV-2) ancestral strains and emerging variants of concern (VoCs) [6–8], and the potential differences between vaccine platforms, hindered the endorsement for dose fractionation of COVID-19 vaccines [9, 10]. Nevertheless, a half-dose of the original Moderna vaccine has been recommended for the booster dose for adults who are not moderately and severely immunocompromised [11].

Here, we conducted a systematic review and meta-analysis of phase I/II trials that reported dose-finding results of immunogenicity and safety profiles for COVID-19 vaccines. As our primary outcome, we estimated the pooled dose-response relationship of neutralizing antibodies (nAbs) against the ancestral strain. We then used the dose-response relationship to project the potential vaccine efficacy of fractional doses against infections of the ancestral strain and VoCs using a hypothesized relation between nAbs and protection [12, 13]. We also reviewed the differences in seroconversion of nAbs, T cell-mediated immune responses, and safety profile between fractional and standard dose (i.e., doses used for final products or phase III trials) groups, to further assess the differences in immunogenicity and safety after receiving fractional and standard doses.

## Methods

### Search strategy and study selection

We searched peer-reviewed publications on clinical trials of SARS-CoV-2 vaccines in PubMed on 9 December 2021. We searched with the following terms: (SARS-CoV-2 OR COVID-19) AND (vaccine AND dose) AND (antibod* OR immun*) (detailed search terms in Additional file 1: Table S1 and Additional file 2) [6, 13–63]. We included dose-escalation studies that reported safety, neutralizing antibodies (nAbs, which were measured by plaque reduction neutralization test, 50% reduction, PRNT_50_ and/or surrogate virus neutralization test, sVNT), and/or T cell-mediated immunity among healthy individuals received SRAS-CoV-2 vaccines (Additional file 1: Tables S2–S4). We excluded (1) studies that did not report immunological response or only reported binding antibody; (2) studies without dose-escalation; (3) studies on non-human hosts; (4) studies on participants with specific health conditions (e.g., cancer, organ transplantation) or pregnancy; (5) studies specifically designed for hybrid immunity (i.e., natural infection or heterogenous vaccinations); and (6) reviews or commentaries (Additional file 1: Fig. S1). We assessed the quality of included studies using the Cochrane Risk of Bias tool 2.0 for randomized trials [46] (Additional file 1: Fig. S2).

### Data extraction and processing

Two reviewers (BY and XH) independently screened the titles and full texts of articles according to the inclusion and exclusion criteria. For each included study, we extracted relevant information of the vaccines and participants onto a standardized form, which includes vaccine name, manufacture, platform, dose fraction, vaccination and sampling schedule, age group, and sizes of vaccinated subjects. Dose fraction (*F*_*i*, *j*_) was defined, for each study (*j*), as the ratio of each examined dose group (*d*_*i*, *j*_; *i* denotes the dose) and the standard dose (*d*_*ref*, *j*_, defined as the dose selected for the approved vaccine product or phase III trials):

1 $${F}_{i,j}=\frac{d_{i,j}}{d_{ref,j}}$$

### Differences in seroconversion of neutralizing antibodies after fractional and standard dose

We compared the proportion of seroconversion to nAbs against ancestral strains after receiving fractional doses compared with the standard dose group, where seroconversion was predefined by each study as at least fourfold increase in nAbs and/or changing from negative to positive after vaccinations (details about definitions for positive threshold and seroconversion were shown in Additional file 1: Table S3). We chose to estimate the pooled risks ratio of seroconversion (RR, i.e., the ratio of proportion of seroconversion between fractional and standard dose groups) over the pooled proportion of seroconversion (i.e., the proportion of seropositive among all investigated participants in each dose group), to minimize the impacts of measurement variations between laboratories. Sample size and the number of seroconverted participants were extracted for each dose group, which were then used to estimate the pooled log RR of seroconversion between fractional and standard dose groups using random effects (RE) model, stratified by vaccine type. We fitted mixed effects meta-regressions to assess the effects of the vaccine platform and dose fractions on seroconversion, after accounting for age group and assay methods.

### Dose-response relationship of neutralizing antibodies

For each study *j*, we extracted the mean (*μ*_*i*, *j*_) and standard deviations (*σ*_*i*, *j*_) of nAbs titers in different dose groups *i*; if not reported, we estimated *μ*_*i*, *j*_ and *σ*_*i*, *j*_ from (1) individual data points or (2) median, interquartile (IQR), and sample sizes [64]. We then standardized the vaccine-induced nAbs level (*z*_*i*, *j*_) using the nAbs measured in convalescent sera (*μ*_*c*, *j*_) for each study:

2 $${z}_{i,j}=\frac{\mu_{i,j}}{\mu_{c,j}}$$

We summarized standardized nAbs among different dose groups (i.e., fractional, standard, and higher dose groups) at different time points (i.e., days after 1 or 2 doses). To quantify the non-linear dose-response relationship of vaccination (log_2_*F*_*i*, *j*_) and the standardized nAbs (*z*_*i*, *j*_), we fitted a generalized additive model (GAM; Additional file 1: Table S5) that accounted for the vaccine platform (*V*), vaccine schedule (i.e., total dosages *D* and days after full vaccination *T*_*i*, *j*_), age group (*A* = children, adult, or elderly), and antigen used for neutralizing assay (*M* = live or pseudo virus):

3 $${\log}_2{z}_{i,j}={\beta}_0+{\beta}_Ts\left({T}_{i,j}\right)+{\beta}_Fs\left({\log}_2{F}_{i,j}\right)+{\beta}_V{V}_j+{\beta}_D{D}_{i,j}+{\beta}_A{A}_{i,j}+{\beta}_M{M}_{i,j}$$

*s*(.) denotes the thin plate spline term. With estimates from equation 3, we predicted the standardized nAbs (assuming measured by live virus and in adults; same for the following) against SARS-CoV-2 ancestral strain 14 days after fully vaccinated (i.e., 1 dose for vector and 2 for the rest) with fractional doses (*F*_*i*, *j*_) for different vaccine platforms. We validated our model predictions and raw data and performed ten-fold cross-validation (Additional file 1: Fig. S3 and Table S6).

### Vaccine efficacy predicted from neutralizing antibodies

We applied the established correlation of protection (CoP) of standardized nAbs [12, 13] to predict the dose-fractioning vaccine efficacy (Φ_*i*_) against symptomatic infections of SARS-CoV-2 ancestral strain for different vaccine platforms (*V*):

4 $${\Phi}_{i,V,E}=\frac{1}{1+{e}^{-{k}_E{\log}_{10}\frac{z_{i,V}}{z_{50,E}}}}$$

We obtained the log-transformed 50% protective efficacy (log_10_*z*_50, *E*_) and steepness parameter *k*_*E*_ for both symptomatic and severe infection from the previous study [12]. *z*_*i*, *V*_ is the standardized nAbs at 14 days after fully vaccinated of fractional doses (*F*_*i*, *j*_) for each vaccine platform, which was estimated from equation 3 with coefficients shown in Additional file 1: Table S5.

We used previously reported [6, 13] fold of reduction (*δ*_*S*_; Additional file 1: Table S7) in nAbs to estimate the level of standardized nAbs (*δ*_*S*_*z*_*i*, *V*_) against the variant *S*, which was then applied to equation 4 to predict the vaccine efficacy of dose fractioning against infections of SARS-CoV-2 VoCs. To validate our predicted vaccine efficacy against VoCs, we compared the predicted vaccine efficacy against symptomatic infections after standard dose and observations (Additional file 1: Table S8) reported previously by Pearson correlation. Standard doses were used for comparison since there were no empirical data regarding half-dose.

### T cell responses

Since assays and measurements used for T cell-mediated responses vary across studies, we reviewed if T cell responses elicited by dose-fractioning vaccines (1) would be higher than that at a pre-vaccination level and (2) would be lower than that elicited by the standard dose vaccine within the same study. Briefly, we extracted the mean (${\overline{x}}_{i,j,k}$; log-transformed if originally measured in log-scale; same for SE), standard error (SE, ${\hat{\sigma}}_{{\overline{x}}_{i,j,k}}$), and sample size (*n*_*i*_) of specific measurement *k* for T cell responses for each dose group or reference group (i.e., pre-vaccination or post standard dose vaccination) *i* in study *j*. Specific measurement (*k*) includes T cell types (i.e., CD4+ or CD8+) and/or cytokines for T helper type 1 (Th1, including interferon-*γ* (IFN- *γ*), tumor necrosis factor (TNF- *α*) and interleukin-2 (IL-2)) and T helper type 2 (Th2, including IL-4, IL-5, IL-13). If mean and SE were not reported, we estimated these metrics from individual original data points or median, IQR and sample sizes [64]. We determined the statistical significance of the difference in (log-)means (Δ_*i*, *j*, *k*_) assuming it follows a normal distribution.

5 $${\overline{\Delta}}_{i,j,k}={\overline{x}}_{i,j,k}-{\overline{x}}_{ref,j,k}$$

6 $${\hat{\sigma}}_{{\overline{\Delta}}_{i,j,k}}=\sqrt{\frac{{{\hat{\sigma}}_{{\overline{x}}_{i,j,k}}}^2}{n_i}+\frac{{{\hat{\sigma}}_{{\overline{x}}_{ref,j,k}}}^2}{n_{ref}}}$$

### Safety

We compared the safety profiles after receiving fractional dose compared with the standard dose group. We extracted the sample size and the number of adverse events (AEs, i.e., solicited local and/or systemic events, unsolicited events, and any AEs) for each dose group. Individual manifestations within each AE category were extracted and assessed. We estimated the pooled log RR of experiencing AEs between fractional and standard dose groups using the RE model and stratifying by specific AE and vaccine platform. We calculated the I^2^ to measure the heterogeneity of the included estimates. We also repeated the above analysis for the higher dose group, which results can be found in our data repository.

## Results

In total 1733 records were returned from PubMed search with 44 duplicates. After title and abstract screening, 136 records were eligible for full-text screen (Additional file 1: Fig. S1). Thirty-eight studies were included in the analyses [15–17, 19, 21–26, 28–32, 36–38, 40–43, 45, 48, 50–63], among which inactivated vaccines (29%, *n* = 11) were studied the most, followed by protein subunit (“subunit” hereafter; 26%, *n* = 10), mRNA (24%, *n* = 9), non-replicating viral vector (“vector” hereafter; 13%, *n* = 5) and others (Additional file 1: Fig. S1 and Table S2). We found overall low risks of bias of the included studies, except that seven adopted the non-randomized, and non-double-blinded design (Additional file 1: Fig. S2) [15, 28, 29, 37, 43, 48, 63].

### Seroconversion of neutralizing antibodies after fractional doses

**Fig. 1:**
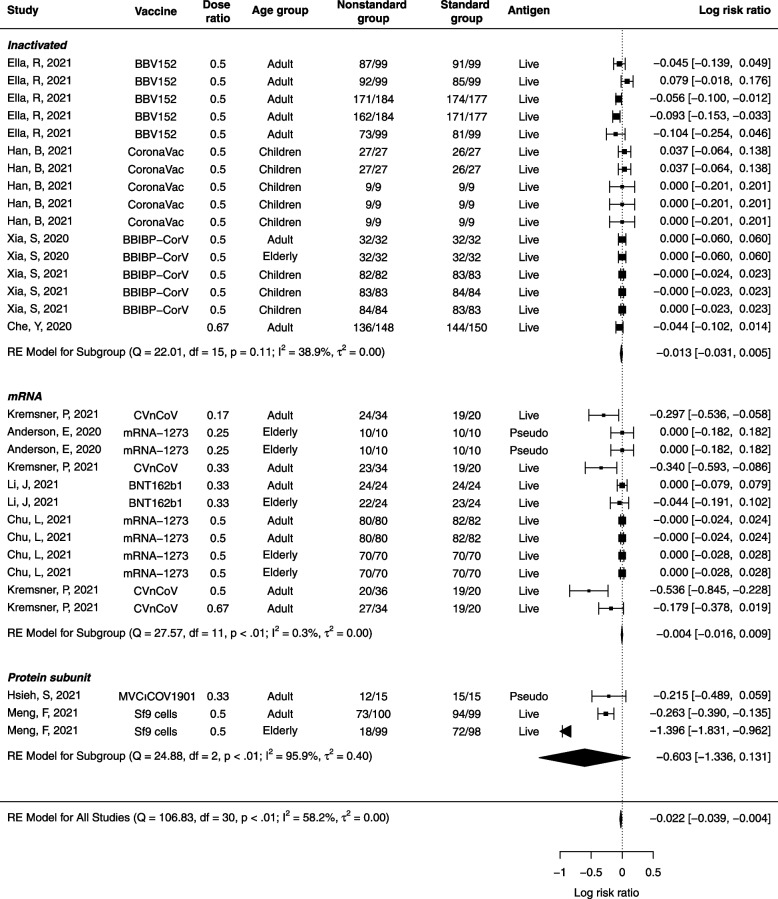
Pooled risk ratio (in log scale) of seroconversion between fractional and standard dose groups of COVID-19 vaccines. Number of seroconversion individuals and sample sizes were shown for the standard and nonstandard groups, respectively

We estimated the pooled RR of the seroconversion against ancestral strains among individuals who completed fractional and standard dose from 14 studies of 9 vaccines (Fig. 1). The probability of seroconversion to ancestral strains was 2.1% (95% confidence interval (CI) 0.4% to 3.6%; *I*^2^= 52.0%, *P*-value < 0.01) lower among individuals with fractional doses compared to standard doses within the same trial. However, we found no association between dose fractionation (1.4%, 95% CI, − 20.4% to 29.3% per fold increase in dose) and seroconversion proportions between lower and standard dose groups after accounting for vaccine platform, age group, and assay methods (i.e., live or pseudo virus) (Additional file 1: Table S9).

### Dose-relationship of neutralizing antibodies and predicted vaccine efficacy

**Fig. 2:**
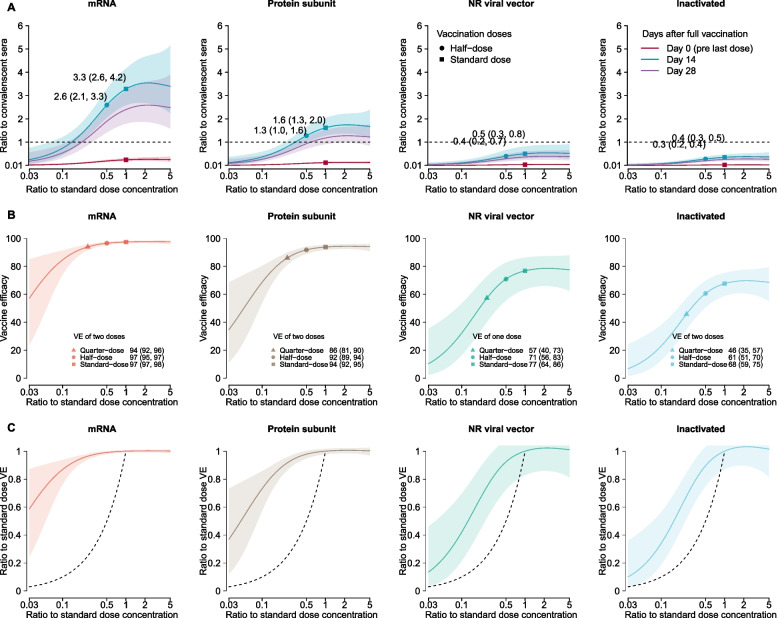
Dose-response relationship of neutralizing antibodies (nAbs) and vaccine efficacy (VE) against ancestral strains induced by COVID-19 vaccines. A 2-dose schedule was assumed for RNA, protein subunit, and inactivated vaccines, while 1-dose schedule was assumed for non-replicating viral vectors (as suggested by the included trials). **A** Dose-response relationship of nAbs against ancestral strains. nAbs were standardized as the ratio to the convalescent sera. Dashed horizontal line indicates the average level of nAbs against ancestral strains in convalescent sera. **B** Dose-response relationship of predicted vaccine efficacy against symptomatic infections of ancestral strains. **C** Association between reduction in vaccine efficacy and dose fractionation. Reductions in vaccine efficacy were measured as the ratio between vaccine efficacy against symptomatic infections of ancestral strains between fractional and standard dose groups

Twenty-four studies reported nAbs against live (*n* = 20) and/or pseudo (*n* = 7) ancestral viruses from both post-vaccination and convalescent sera (Additional file 1: Figs. S4–S6 and Table S3). We estimated that prime with one half-dose would elicit less than 10% of nAbs in convalescent sera, while prime-boost with two half-doses elicited higher nAbs than a single standard dose across all platforms (Fig. 2A and Additional file 1: Fig. S3–S7).

We estimated that two half-dose mRNA vaccines would elicit 2.6 (95% CI, 2.1 to 3.3, measured on day 14)-fold of the nAbs against the ancestral strain in convalescent sera (Fig. 2A), which is expected to prevent 97% (95% CI, 95% to 97%) of symptomatic infections of the ancestral strains, respectively (Fig. 2B). Whereas two half-dose inactivated vaccines would elicit 0.28 (95% IC 0.20 to 0.37)-fold of nAbs against the ancestral strains in convalescent sera, corresponding to 61% (95% CI, 51% to 70%) and 95% (95% CI, 92% to 96%) efficacy against symptomatic and severe infections of the ancestral strains, respectively. Overall, our predictions suggested that the reduction in vaccine efficacy was smaller than dose fractionation across all vaccine platforms (Fig. 2C); half-doses may provide more than half of protection efficacy of standard doses.

**Fig. 3:**
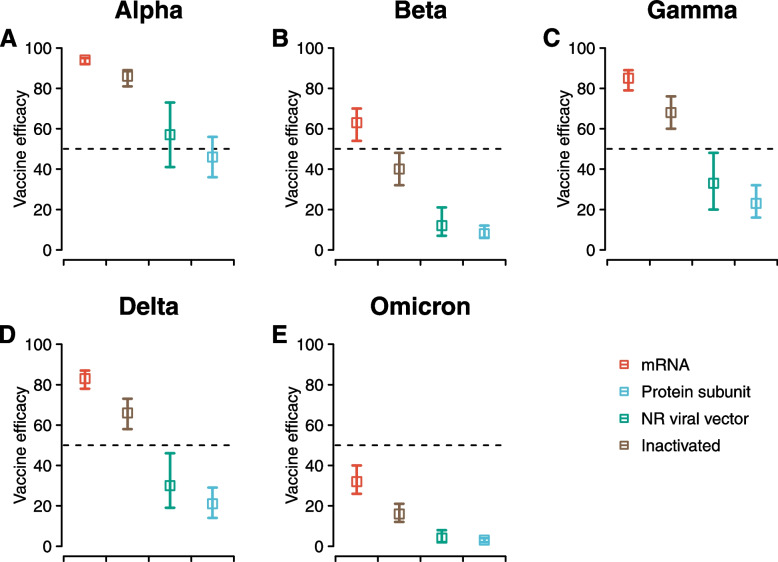
Predicted vaccine efficacy against SARS-CoV-2 variants of concern after fully vaccinated with half-dose vaccines. Vaccine efficacy against symptomatic infections after full vaccinations (i.e., one dose non-replicating viral vector and two doses for the rest) of half-dose is shown, with the complete dose-dependent effectiveness shown in Additional file 1: Fig. S8. A-E for Alpha, Beta, Gamma, Delta, and Omicron

Further incorporating the reported fold reduction in of vaccine-induced nAbs against VoCs (Additional file 1: Table S7) [6, 13], we projected that two half-dose mRNA vaccines would confer the highest efficacy against symptomatic infections of VoCs (94%, 95% CI, 92% to 95% against Alpha, 63%, 54% to 70% against Beta, 85%, 79% to 89% against Gamma, 83%, 78% to 87% against Delta, and 32%, 26% to 40% against Omicron), followed by subunit, vector and inactivated vaccines (Fig. 3 and Additional file 1: Fig. S8). Our predicted efficacy against symptomatic infections of VoCs for standard dose highly correlated (Pearson correlation 0.705, *p*-value < 0.01; Fig. S9) with empirical data [14, 18, 27, 33–35, 39, 44, 47, 49, 65], while we were not able to validate predictions for fractional doses due to lack of data. Results from ten-fold validations further supported our model fitting (Additional file 1: Table S6).

### T cell responses after fractional doses

**Fig. 4:**
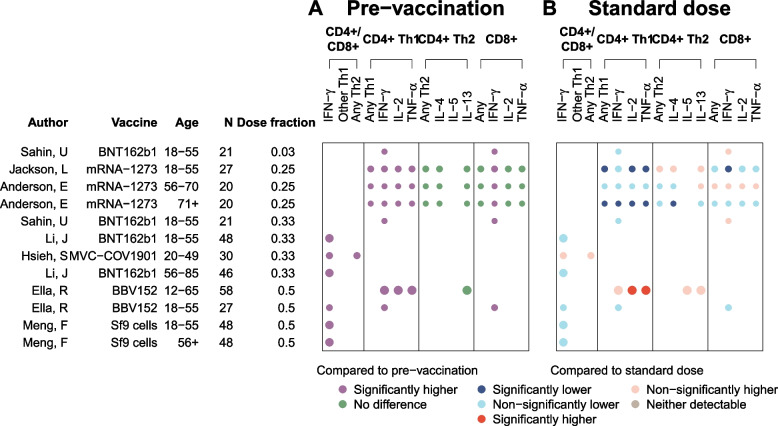
Comparison of T cell responses against the ancestral strains elicited by dose fractioning of COVID-19 vaccines. The size of dots represents the total sample sizes of the standard and non-standard dose groups. **A** Compared to pre-vaccination. If the mean and 95% CI of the difference in mean T cell levels before and after the fractional doses were all greater than 0, we determined T cell responses were significantly higher between the groups. **B** Compared to people who received standard doses. If the mean and 95% CI of the difference in mean T cell levels between the fractional and standard dose groups were all greater or less than 0, we determined T cell responses were significantly higher or lower than that elicited by the standard dose

We first reviewed whether T cell-mediated immune responses elicited by dose fractioning vaccines would be higher than the pre-vaccination level. All 7 studies of 5 vaccines reported a significant increase in SARS-CoV-2 specific CD4+/CD8+ or CD4+ T helper type 1 (Th1) responses after vaccinated with fractional doses compared to pre-vaccination (Fig. 4A), which were all biased to Th1 cells.

We then reviewed whether T cell responses would be lower than that elicited by the standard dose vaccine. Three vaccines (BNT162b1 [32, 43], MVC-COV1901 [28], and Sf9 cells [36]) reported that dose fractionation elicited a similar level of CD4+ and/or CD8+ T cells compared to the standard dose (Fig. 4B). Quarter-dose of mRNA-1273 [15, 29] was reported to induce significantly lower CD4+ Th1 cells compared to the standard dose, while half-dose of BBV152 were reported to induce significantly higher Th1 cytokines in one of two trials. We also compared the cellular responses between standard and higher dose groups and found no evidence for a dose-dependent relationship for 7 out of 9 vaccines (Additional file 1: Fig. S10).

### Safety profiles after fractional doses

We reviewed the safety profile for 34 studies and found that, compared to the standard dose group, people in the fractional dose groups tended to experience adverse events at similar or lower frequency (Fig. S11–16). Particularly, the risk of experiencing solicited, and unsolicited adverse events were 9.5% (95% CI, 3.9% to 14.8 %) and 24.4% (3.9% to 41.1%) lower in individuals who received a factional dose of mRNA vaccines compared to standard doses (Fig. S14–15). One inactivated (BBIBP-CorV in children [56]) and two subunit (NVX- CoV2373 and Livzon in adults [23, 45]) vaccines reported a higher risk of solicited systemic reactions in groups that received a lower dose than the standard dose.

## Discussion

We reported the pooled dose-response relationship of nAbs against the ancestral strains using estimates from phase I/II studies. Our findings suggested that vaccine-induced nAbs varied substantially across dose fractions, number of dosages, and vaccine platforms. For vaccine platforms (e.g., mRNA and subunit) which standard doses could elicit higher nAbs levels than convalescent sera, fractionation of prime-boost doses could induce robust nAbs against the ancestral strains and similar seroconversion proportion with standard doses. nAbs induced by fractional vaccines of mRNA and subunit were predicted to confer ≥ 65% efficacy against symptomatic infections of SARS-CoV-2 variants, except for Beta and Omicron. Fractionation of vaccine doses seemed to be safe and induce robust Th1-biased T cell responses that were similar to standard doses except for mRNA-1273.

We found that dose fractionation of COVID-19 vaccines would induce, though lower than standard doses, detective nAbs against ancestral strains. Based on previously established CoP [12, 13, 66], these nAbs may confer reasonable protection (i.e., > 50%) against symptomatic infections of ancestral strains, but not the subsequent VoCs, especially Omicron. Previous modeling study suggested that dose fractionation could be a cost-effective strategy in low-income countries, if vaccination could confer at least 50% of protection against symptomatic infections of variants with low or moderate transmissibility (i.e., basic reproduction number *R*_0_ < 5) [67]. Given these findings, our results of nAbs and vaccine efficacy predictions suggested that dose fractionation could have been a cost-effective strategy to control the emergence of some early VoCs (e.g., Alpha and Delta), but not for the currently circulating Omicron given the significant immunity breakthrough [6, 7] and higher transmissibility [68]. With the development of reformulated COVID-19 vaccines using the Omicron variant, fractionation of vaccines in the subsequent booster dose allocation may still be effective yet further investigations are needed.

While there is no established correlate of protection against severe COVID, we found that fractional doses of most studied vaccines could induce detective and likely robust T cell responses, which may contribute to protection against severe outcomes given that SARS-CoV-2 specific T cells could broadly cross-react to a range of VoCs (including Omicron) and were associated with better outcomes [69, 70]. Therefore, dose fractionation of COVID-19 vaccines might still be able to avert a considerable number of hospitalizations and deaths, even with the emergence of new variants with higher rates of breakthrough infections.

We were not able to assess the durability of the immune responses elicited by fractional doses of COVID-19 vaccines, as most trials reported limited follow-up that was typically just one month after vaccination. Therefore, our efficacy estimates may only be indicative for short-term protection. Waning SARS-CoV-2 specific aAbs, T cells, and vaccine efficacy (against both ancestral and VoCs) may be expected, as suggested by evidence from individuals receiving standard doses after 6 months [7, 71–73]. For standard doses, both homogeneous and heterogenous boosters could substantially increase nAbs and vaccine efficacy against VoCs [8], while such data were lacking for fractional doses.

To minimize the impacts of measurement variations between laboratories, we compared the differences in seroconversion of nAbs and T cell responses within each trial and quantified the dose-relationship using nAbs that were standardized to convalescent sera. Calibration to recommended international standard may further reduce the between laboratory variations, which was, however, not reported by the included trials.

We did not look at the nAbs induced by individual vaccine manufacturers due to limited data, while we found consistent seroconversion proportion and dose-relationship within platform (Additional file 1: Fig. S3–S7). Nevertheless, disparities in nAbs levels and durability were reported for individual vaccines from the same platform (e.g., mRNA-1273 vs. BNT162b1 vaccines [9]). Of not, dose-response relationship may vary across vaccine platforms, while we do not have sufficient data for further investigations.

Our results indicated that nAbs and the projected protections after two half-doses were higher than that after one standard dose. Therefore, two half-doses could make more efficient usage of the limited antigen (especially early in the pandemic) and potentially save more lives compared to one standard dose, despite for the higher logistical cost for vaccine administration [67].

We found that the safety of fractionation of vaccine doses seems to be non-inferior to that of the standard doses. However, our pooled safety estimates may be underpowered to detect rare safety events, as most of the included studies were phase I and II trials that were designed with small sample sizes.

Our study only focused on the immunogenicity and safety and the projected efficacy of dose fractionation of COVID-19 vaccines, and therefore findings should be interpreted within this scope. The projected VE under the smallest fractional doses (e.g., 10% to 30%) may suffer greater uncertainties from smaller sample sizes and edge effects of GAM estimations, despite that several studies reported similar or slightly lower seroconversion risk in these low dose groups. In addition, some of the vaccine effectiveness estimates we used to validate the projected vaccine efficacies of fractional doses were estimated in observational studies, which may also be subject to a number of biases. Therefore, endorsement of dose fractionation of vaccines by regulatory agencies would likely need stronger efficacy data, and other considerations would include the evolving supply situation, logistics restrictions, and vaccine communications.

## Conclusions

To summarize, fractionation of vaccine doses, especially mRNA and protein subunit vaccines, are safe and would induce antibody and T cell responses that likely confer a reasonable level of protection against severe infections of SARS-CoV-2 ancestral and VoCs up to Omicron. The use of vaccines with lower antigen content earlier in the pandemic might have been an efficient approach to save even more lives, while further clinical investigation of fractional booster doses would certainly be worthwhile.

## Supplementary Information

**Additional file 1: Table S1.** Search strategy and number of articles identified in each step. **Table S2.** Summary of 38 studies that were included for systematic review analysis. **Table S3.** Summary of 38 studies that were included for analyses of seroconversion and dose-response relationship of neutralizing antibodies. **Table S4.** Summary of 17 studies that are included for cell-mediated response analysis by vaccine type. **Table S5.** Factors associated with neutralizing antibody responses after receiving different fractional doses of vaccinations. **Table S6.** Cross-validation of general additive model for dose-response relationship of neutralizing antibody after fractional doses. **Table S7.** Fold of reduction in neutralizing antibodies against variants of concerns. **Table S8.** Vaccine effectiveness against infections of variants of concern for standard dose. **Table S9.** Factors associated with risk of experiencing seroconversion of neutralizing antibodies after receiving non-standard and standard doses. **Fig. S1.** Flowchart of literature search and screening. **Fig. S2.** Risk of bias of 39 included studies. **Fig. S3.** Model predictions of dose-response relationship of neutralizing antibodies (nAbs) against ancestral strains introduced by COVID-19 vaccines. **Fig. S4.** Standardized neutralizing antibodies (nAbs) introduced by COVID-19 vaccines on day 0 since the complete vaccination. **Fig. S5.** Standardized neutralizing antibodies (nAbs) introduced by COVID-19 vaccines on day 14 since the complete vaccination. **Fig. S6.** Standardized neutralizing antibodies (nAbs) introduced by COVID-19 vaccines on day 28 or later since the complete vaccination. **Fig. S7.** Associations between time since complete vaccination and the standardized neutralizing antibodies (nAbs) against the ancestral strains elicited by fractioning dose of COVID-19 vaccines. **Fig. S8.** Dose-relationship between dose fractionation and predicted vaccine efficacy against symptomatic infections of variants of concern. **Fig. S9.** Correlation between predicted and observed vaccine efficacy against variants of concern for standard dose of COVID-19 vaccines. **Fig. S10.** Comparison of T-cell responses against the ancestral strains elicited by higher doses of COVID-19 vaccines. **Fig. S11.** Comparison of safety after vaccinated with lower doses (a) and higher doses (b) to standard doses of SARS-CoV-2 vaccines. **Fig. S12.** Pooled risk ratio (in log scale) of experiencing solicited local adverse events after vaccinated with fractional and standard dose groups. **Fig. S13.** Pooled risk ratio (in log scale) of experiencing solicited systemic adverse events after vaccinated with fractional and standard dose groups. **Fig. S14.** Pooled risk ratio (in log scale) of experiencing any solicited adverse events after vaccinated with fractional and standard dose groups. **Fig. S15.** Pooled risk ratio (in log scale) of experiencing any unsolicited adverse events after vaccinated with fractional and standard dose groups. **Fig. S16.** Pooled risk ratio (in log scale) of experiencing any adverse events after vaccinated with fractional and standard dose groups.

**Additional file 2.** Preferred reporting items for systematic reviews and meta-analyses (PRISMA) list.

## Data Availability

All data were collected from publicly available literatures, with detailed description in the “Methods” section and Additional file 1. Data used for the analysis can be assessed at [74]. The authors declare that all codes for analyzing the data are made available at https://github.com/byyangyby/fractional_dose_review.

## Abbreviations

AEs: Adverse events
CI: Confidence interval
CoP: Correlation of protection
COVID-19: Coronavirus disease 2019
GAM: Generalized additive model
IFN: Interferon
IL: Interleukin
IQR: Interquartile
nAbs: Neutralizing antibodies
PRNT_50_: Plaque reduction neutralization test, 50% reduction
RE: Random effects
RR: Risk ratio
SARS-CoV-2: Severe acute respiratory syndrome coronavirus 2
SE: Standard error
sVNT: Surrogate virus neutralization test
Th1: T helper type 1
Th2: T helper type 2
TNF: Tumor necrosis factor
VoCs: Variants of concern
