# Supplementary material for: Immunogenicity, efficacy, and safety of SARS-CoV-2 vaccine dose fractionation: a systematic review and meta-analysis
Source: BMC Med. 2022 Oct 25;20:409. doi: 10.1186/s12916-022-02600-0 (PMC9595080; doi:10.1186/s12916-022-02600-0)
Supplement: Supplementary file 1 — Additional file 1: Table S1. Search strategy and number of articles identified in each step. Table S2. Summary of 38 studies that were included for systematic review analysis. Table S3. Summary of 38 studies that were included for analyses of seroconversion and dose-response relationship of neutralizing antibodies. Table S4. Summary of 17 studies that are included for cell-mediated response analysis by vaccine type. Table S5. Factors associated with neutralizing antibody responses after receiving different fractional doses of vaccinations. Table S6. Cross-validation of general additive model for dose-response relationship of neutralizing antibody after fractional doses. Table S7. Fold of reduction in neutralizing antibodies against variants of concerns. Table S8. Vaccine effectiveness against infections of variants of concern for standard dose. Table S9. Factors associated with risk of experiencing seroconversion of neutralizing antibodies after receiving non-standard and standard doses. Fig. S1. Flowchart of literature search and screening. Fig. S2. Risk of bias of 39 included studies. Fig. S3. Model predictions of dose-response relationship of neutralizing antibodies (nAbs) against ancestral strains introduced by COVID-19 vaccines. Fig. S4. Standardized neutralizing antibodies (nAbs) introduced by COVID-19 vaccines on day 0 since the complete vaccination. Fig. S5. Standardized neutralizing antibodies (nAbs) introduced by COVID-19 vaccines on day 14 since the complete vaccination. Fig. S6. Standardized neutralizing antibodies (nAbs) introduced by COVID-19 vaccines on day 28 or later since the complete vaccination. Fig. S7. Associations between time since complete vaccination and the standardized neutralizing antibodies (nAbs) against the ancestral strains elicited by fractioning dose of COVID-19 vaccines. Fig. S8. Dose-relationship between dose fractionation and predicted vaccine efficacy against symptomatic infections of variants of concern. Fig. S9. Correlation b [file 12916_2022_2600_MOESM1_ESM.docx]

**Additional File**

**Immunogenicity, efficacy, and safety of SARS-CoV-2 vaccine dose fractionation: a systematic review and meta-analysis**

Bingyi Yang^1^, Xiaotong Huang^1^, Huizhi Gao^1^, Nancy H. Leung^1^, Tim K. Tsang^1,2^, Benjamin J. Cowling^1,2^

**Affiliations:**

1. WHO Collaborating Centre for Infectious Disease Epidemiology and Control, School of Public Health, Li Ka Shing Faculty of Medicine, The University of Hong Kong, Hong Kong, China
2. Laboratory of Data Discovery for Health Limited, Hong Kong Science and Technology Park, New Territories, Hong Kong, China

**Correspondence to:**

Benjamin J. Cowling, ([bcowling@hku.hk](mailto:bcowling@hku.hk)); Bingyi Yang ([yangby@hku.hk](mailto:yangby@hku.hk))

Table of Contents

[Supplementary Tables 4](#_Toc115961921)

[Table S1. Search strategy and number of articles identified in each step. 4](#_Toc115961922)

[Table S2. Summary of 38 studies that were included for systematic review analysis. 5](#_Toc115961923)

[Table S3. Summary of 38 studies that were included for analyses of seroconversion and dose-response relationship of neutralizing antibodies. 9](#_Toc115961924)

[Table S4. Summary of 17 studies that are included for cell-mediated response analysis by vaccine type. 13](#_Toc115961925)

[Table S5. Factors associated with neutralizing antibody responses after receiving different fractional doses of vaccinations. 15](#_Toc115961926)

[Table S6. Cross-validation of general additive model for dose-response relationship of neutralizing antibody after fractional doses. 15](#_Toc115961927)

[Table S7. Fold of reduction in neutralizing antibodies against variants of concerns. 16](#_Toc115961928)

[Table S8. Vaccine effectiveness against infections of variants of concern for standard dose. 17](#_Toc115961929)

[Table S9. Factors associated with risk of experiencing seroconversion of neutralizing antibodies after receiving non-standard and standard doses. 18](#_Toc115961930)

[Supplementary Figures 19](#_Toc115961931)

[Fig. S1. Flowchart of literature search and screening. 19](#_Toc115961932)

[Fig. S2. Risk of bias of 39 included studies. 20](#_Toc115961933)

[Fig. S3. Model predictions of dose-response relationship of neutralizing antibodies (nAbs) against ancestral strains introduced by COVID-19 vaccines. 21](#_Toc115961934)

[Fig. S4. Standardized neutralizing antibodies (nAbs) introduced by COVID-19 vaccines on day 0 since the complete vaccination. 22](#_Toc115961935)

[Fig. S5. Standardized neutralizing antibodies (nAbs) introduced by COVID-19 vaccines on day 14 since the complete vaccination. 23](#_Toc115961936)

[Fig. S6. Standardized neutralizing antibodies (nAbs) introduced by COVID-19 vaccines on day 28 or later since the complete vaccination. 24](#_Toc115961937)

[Fig. S7. Associations between time since complete vaccination and the standardized neutralizing antibodies (nAbs) against the ancestral strains elicited by fractioning dose of COVID-19 vaccines. 25](#_Toc115961938)

[Fig. S8. Dose-relationship between dose fractionation and predicted vaccine efficacy against symptomatic infections of variants of concern. 26](#_Toc115961939)

[Fig. S9. Correlation between predicted and observed vaccine efficacy against variants of concern for standard dose of COVID-19 vaccines. 27](#_Toc115961940)

[Fig. S10. Comparison of T-cell responses against the ancestral strains elicited by higher doses of COVID-19 vaccines. 28](#_Toc115961941)

[Fig. S11. Comparison of safety after vaccinated with lower doses (a) and higher doses (b) to standard doses of SARS-CoV-2 vaccines. 29](#_Toc115961942)

[Fig. S12. Pooled risk ratio (in log scale) of experiencing solicited local adverse events after vaccinated with fractional and standard dose groups. 30](#_Toc115961943)

[Fig. S13. Pooled risk ratio (in log scale) of experiencing solicited systemic adverse events after vaccinated with fractional and standard dose groups. 31](#_Toc115961944)

[Fig. S14. Pooled risk ratio (in log scale) of experiencing any solicited adverse events after vaccinated with fractional and standard dose groups. 32](#_Toc115961945)

[Fig. S15. Pooled risk ratio (in log scale) of experiencing any unsolicited adverse events after vaccinated with fractional and standard dose groups. 33](#_Toc115961946)

[Fig. S16. Pooled risk ratio (in log scale) of experiencing any adverse events after vaccinated with fractional and standard dose groups. 34](#_Toc115961947)

# Supplementary Tables

## Table S1. Search strategy and number of articles identified in each step.

| **Step** | **Search terms** | **Number of results** |
| --- | --- | --- |
| #1 | SARS-CoV-2 OR COVID-19 | 205,618 |
| #2 | vaccine AND dose | 34,113 |
| #3 | antibod* OR immun* | 4,574,287 |
| #4 | #1 AND #2 AND #3 | 1,733 |

*Searches were conducted on 9 Dec 2021 in PubMed.

## Table S2. Summary of 38 studies that were included for systematic review analysis.

| **Reference** | **Vaccine**  **(Manufacturers)** | **Phase** | **Age group**  **(years)** | **Vaccine schedule** | **Dose concentration** | **Safety** |
| --- | --- | --- | --- | --- | --- | --- |
| **Inactivated virus (n = 11, 29%)** | | | | | | |
| Xia, S, 2020, *Lancet Infect Dis [56]* | BBIBP-CorV (Sinopharm (Beijing)) | 1/2 | 18-59,  60-80 | 1 dose;  2 doses, 0 days apart;  2 doses, 14 days apart;  2 doses, 21 days apart;  2 doses, 28 days apart | 2$ug$, 4 $ug$, 8 $ug$ | Available |
| Xia, S, 2021, *Lancet Infect Dis* [57] | BBIBP-CorV (Sinopharm (Beijing)) | 1/2 | 3-5,  6-12,  13-17 | 3 doses, 28 days apart | 2 $ug$, 4 $ug$, 8 $ug$ | Available |
| Ella, R, 2021 (21:637-46),  *Lancet Infect Dis [22]* | BBV152 (Covaxin) | 1 | 18-55 | 2 doses, 14 days apart | 3 $ug$, 6 $ug$ | Available |
| Ella, R, 2021 (21:950-61),  *Lancet Infect Dis* [21] | BBV152 (Covaxin) | 1/2 | 12-65,  18-55 | 2 doses, 28 days apart | 3 $ug$, 6 $ug$ | Available |
| Che, Y, 2020, *Clin Infect Dis* [17] | Chinese Academy of Medical Sciences | 2 | 18-59 | 2 doses, 14 days apart;  2 doses, 28 days apart | 100EU, 150EU | Available |
| Han, B, 2021, *Lancet Infect Dis* [26] | CoronaVac (Sinovac) | 1/2 | 3-5,  3-17,  6-11,  12-17 | 2 doses, 28 days apart | 1.5 $ug$, 3 $ug$ | Available |
| Wu, Z, 2021, *Lancet Infect Dis* [54] | CoronaVac (Sinovac) | 1/2 | 60- | 2 doses, 28 days apart | 3 $ug$, 6 $ug$, 1.5 $ug$ | Available |
| Zhang, Y, 2021, *Lancet Infect Dis* [60] | CoronaVac (Sinovac) | 1 | 18-59 | 2 doses, 14 days apart;  2 doses, 28 days apart | 3 $ug$, 6 $ug$ |  |
| Pan, H, 2021, *Chin Med J* [40] | KCONVAC  (Minhai BioTech) | 1/2 | 18-59 | 2 doses, 14 days apart;  2 doses, 28 days apart | 5 $ug$, 10 $ug$ | Available |
| Xia, S, 2020, *JAMA* [55] | Sinopharm (Wuhan) | 1/2 | 18-59 | 2 doses, 14 days apart;  2 doses, 21 days apart;  3 doses, 28 days apart | 2.5 $ug$, 5 $ug$ , 10 $ug$ | Available |
| Guo, W, 2021, *EClinicalMedicine* [25] | Sinopharm (Wuhan) | 1/2 | 18-59,  60- | 1 dose;  2 doses, 14 days apart;  2 doses, 21 days apart;  2 doses, 28 days apart;  3 doses, 28 days apart | 5 $ug$, 10 $ug$ , 2.5 $ug$ | Available |
| **Protein subunit (n = 10, 26%)** | | | | | | |
| *Goepfert*, P, 2021, *Lancet Infect Dis* [24] | Sanofi/GSK | 1/2 | 18-49,  50- | 2 doses, 21 days apart | 1.3 $ug$, 2.6 $ug$ | Available |
| Chappell, K, 2021, *Lancet Infect Dis* [16] | Sclamp (Queensland) | 1 | 18-55 | 1 dose;  2 doses, 28 days apart | 5 $ug$, 15 $ug$, 45 $ug$ | Available |
| Hsieh, S, 2021, *EClinicalMedicine* [28] | MVCCOV1901 (Medigen) | 1 | 20-49 | 2 doses, 28 days apart | 5 $ug$, 15 $ug$, 25 $ug$ | Available |
| Formica, N, 2021, *PLoS Med* [23] | NVX-CoV2373 (Novavax) | 2 | 18-55,  18-59,  60-84 | 1 dose;  2 doses, 21 days apart | 5 $ug$, 25 $ug$ | Available |
| Keech, C, 2020, *N Engl J Med* [30] | NVX-CoV2373 (Novavax) | 1 | 18-59 | 1 dose;  2 doses, 21 days apart | 5 $ug$, 25 $ug$ | Available |
| Richmond, P, 2021, *Lancet* [41] | SCB-2019 (Clover) | 1 | 18-54 | 2 doses, 21 days apart | 3 $ug$, 9 $ug$ , 30 $ug$ | Available |
| Meng, F, 2021,  *Signal Transduct Target Ther* [36] | Sf9 cells  (West China Hospital) | 1/2 | 18-55,  56- | 2 doses, 21 days apart;  2 doses, 28 days apart;  3 doses, 14 days apart;  3 doses, 28 days apart | 20 $ug$, 40 $ug$ | Available |
| Shu, Y, 2021, *Chin Med J* [45] | V-01  (Livzon Mabpharm) | 2 | 18-59,  60- | 1 dose;  2 doses, 21 days apart | 50 $ug$, 10 $ug$ , 25 $ug$ | Available |
| Zhang, J, 2021,  *Emerg Microbes Infect* [59] | V-01  (Livzon Mabpharm) | 1 | 18-59, 60- | 2 doses, 21 days apart | 10 $ug$, 25 $ug$ , 50 $ug$ | Available |
| Yang, S, 2021, *Lancet Infect Dis* [58] | ZF2001 (Anhui Zhifei) | 1/2 | 18-59 | 2 doses, 30 days apart;  3 doses, 30 days apart | 25 $ug$, 50 $ug$ | Available |
| **RNA (n = 9, 24%)** | | | | | | |
| Li, J, 2021, *Nat Med* [32] | BNT162b1 (Pfizer/BioNTech) | 1 | 18-55,  56-85 | 2 doses, 21 days apart | 10 $ug$, 30 $ug$ | Available |
| Mulligan, M, 2020, *Nature* [38] | BNT162b1 (Pfizer/BioNTech) | 1/2 | 18-55 | 1 dose;  2 doses, 21 days apart | 10 $ug$, 30 $ug$ , 100 $ug$ | Available |
| Sahin, U, 2020, *Nature* [43] | BNT162b1 (Pfizer/BioNTech) | 1/2 | 18-55 | 1 dose;  2 doses, 21 days apart | 1u $ug$ 10 $ug$ , 30 $ug$, 50 $ug$,  60 $ug$ | Not available |
| Walsh, E, 2020, *N Engl J Med* [50] | BNT162b1, BNT162b2 (Pfizer/BioNTech) | 1 | 18-55,  56-85 | 2 doses, 21 days apart | 10 $ug$, 20 $ug$ , 30 $ug$ | Available |
| Walter, E, 2021, *N Engl J Med* [51] | BNT162b2 (Pfizer/BioNTech) | 1/2 | 5-11 | 2 doses, 21 days apart | 10 $ug$, 20 $ug$ | Available |
| Kremsner, P, 2021,  *Wien Klin Wochenschr* [31] | CVnCoV (Curevac) | 1 | 18-60 | 2 doses, 28 days apart | 2 $ug$, 4 $ug$,  6 $ug$, 8 $ug$,  12 $ug$ | Available |
| Anderson, E, 2020, *N Engl J Med* [15] | mRNA-1273 (Moderna) | 1 | 18-55,  56-70,  71- | 2 doses, 28 days apart | 25 $ug$, 100 $ug$ | Available |
| Chu, L, 2021, *Vaccine* [19] | mRNA-1273 (Moderna) | 2 | 18-54,  55- | 2 doses, 28 days apart | 50 $ug$, 100 $ug$ | Available |
| Jackson, L, 2020, *N Engl J Med* [29] | mRNA-1273 (Moderna) | 1 | 18-55 | 2 doses, 28 days apart | 25 $ug$, 100 $ug$ , 250 $ug$ | Available |
| **Non-replicating viral vector (n = 5, 13%)** | | | | | | |
| Sadoff, J, 2021, *N Engl J Med* [42] | Ad26.COV2.S (Janssen) | 1/2 | 18-55,  56- | 1 dose;  2 doses, 21 days apart | 5$\times$10^10^ VP , 10$\times$10^10^ VP | Available |
| Wu, S, 2021, *Lancet Infect Dis* [53] | Ad5-nCoV (AMMS) | 1 | 18- | 1 dose;  2 doses, 0 days apart | 5$\times$10^10^ VP, 10$\times$10^10^ VP | Available |
| Zhu, F, 2020 (395: 1845-54), *Lancet* [63] | Ad5-nCoV (CanSino) | 1 | 18-60 | 1 dose | 5$\times$10^10^ VP, 10$\times$10^10^ VP, 15$\times$10^10^ VP | Available |
| Zhu, F, 2020 (396: 479-88), *Lancet* [62] | Ad5-nCoV (CanSino) | 2 | 18- | 1 dose | 5$\times$10^10^ VP, 10$\times$10^10^ VP | Available |
| Zhu, F, 2021, *Clin Infect Dis* [61] | Ad5-nCoV (CanSino) | 2 | 56- | 2 doses, 56 days apart | 5$\times$10^10^ VP, 10$\times$10^10^ VP | Available |
| **DNA (n = 2, 5%)** | | | | | | |
| Tebas, 2021, *EClinicalMedicine* [48] | INO-4800 (Inovio) | 1 | 18-50 | 2 doses, 28 days apart | 1 $mg$, 2 $mg$ | Available |
| Momin, T, 2021, *EClinicalMedicine* [37] | ZyCoV-D  (Zydus Cadila) | 1 | 18-55 | 2 doses, 28 days apart | 1 $mg$, 2 $mg$ | Available |
| **Virus-like particle (n = 1, 3%)** |  |  |  |  | |  |
| Ward, B, 2021, *Nat Med* [52] | Plant-based VLP (Medicago) | 1 | 18-55 | 2 doses, 21 days apart | 3.75 $ug$, 7.5 $ug$, 15 $ug$ | Available |

## Table S3. Summary of 38 studies that were included for analyses of seroconversion and dose-response relationship of neutralizing antibodies.

| **Reference** | **Vaccine**  **(Manufacturers)** | **Assay antigen** | **Assay measurement** | **Positive**  **cut-off** | **Seroconversion** | **Seroconversion definition** | **Convalescent sera** |
| --- | --- | --- | --- | --- | --- | --- | --- |
| **Inactivated virus (n = 11, 29%)** | | | | | | | |
| Xia, S, 2020, *Lancet Infect Dis [56]* | BBIBP-CorV (Sinopharm (Beijing)) | Live | MNT_50_ | 1:2 | Available | $\geq$ 4-fold rise | Not available |
| Xia, S, 2021, *Lancet Infect Dis* [57] | BBIBP-CorV (Sinopharm (Beijing)) | Live | MNT_50_ | 1:2 | Available | $\geq$ 4-fold rise | Available |
| Ella, R, 2021 (21:637-46),  *Lancet Infect Dis [22]* | BBV152 (Covaxin) | Live | MNT_50_, PRNT_50_ | Not reported | Available | $\geq$ 4-fold rise | Available |
| Ella, R, 2021 (21:950-61),  *Lancet Infect Dis* [21] | BBV152 (Covaxin) | Live | MNT_50_, PRNT_50_ | 1:20  1:20 | Available | $\geq$ 4-fold rise | Available |
| Che, Y, 2020, *Clin Infect Dis* [17] | Chinese Academy of Medical Sciences | Live | MNT_50_ | 1:4 | Available | Seronegative to seropositive | Not available |
| Han, B, 2021, *Lancet Infect Dis* [26] | CoronaVac (Sinovac) | Live | MNT_50_ | Not reported | Available | Seronegative to seropositive | Not available |
| Wu, Z, 2021, *Lancet Infect Dis* [54] | CoronaVac (Sinovac) | Live | MNT_50_ | 1:8 | Available | Seronegative to seropositive, or  $\geq$ 4-fold rise for baseline seropositive | Not available |
| Zhang, Y, 2021, *Lancet Infect Dis* [60] | CoronaVac (Sinovac) | Live | MNT_50_ | 1:8  1:30 | Available | Seronegative to seropositive, or  $\geq$ 4-fold rise for baseline seropositive | Available |
| Pan, H, 2021, *Chin Med J* [40] | KCONVAC  (Minhai BioTech) | Live,  pseudo | Not reported,  sVNA IC_50_ | 1:4  1:30 | Available | Seronegative to seropositive, or  $\geq$ 4-fold rise for baseline seropositive | Available |
| Xia, S, 2020, *JAMA* [55] | Sinopharm (Wuhan) | Live | PRNT_50_ | Not reported | Available | $\geq$ 4-fold rise | Not available |
| Guo, W, 2021, *EClinicalMedicine* [25] | Sinopharm (Wuhan) | Live | PRNT_50_ | Not reported | Available | $\geq$ 4-fold rise | Not available |
| **Protein subunit (n = 10, 26%)** | | | | | | | |
| Goepfert, P, 2021, *Lancet Infect Dis* [24] | Sanofi/GSK | Live | MNT_50_ | 1:10 | Available | Seronegative to seropositive | Not available |
| Chappell, K, 2021, *Lancet Infect Dis* [16] | Sclamp (Queensland) | Live | MNT_50_ | 1:20 | Not available | -- | Available |
| Hsieh, S, 2021, *EClinicalMedicine* [28] | MVCCOV1901 (Medigen) | Live,  pseudo | Not reported,  sVNA ID_50_ | Not reported | Available | Not reported | Available |
| Formica, N, 2021, *PLoS Med* [23] | NVX-CoV2373 (Novavax) | Live | MNT_50_ | 1:20 | Available | $\geq$ 4-fold rise | Available |
| Keech, C, 2020, *N Engl J Med* [30] | NVX-CoV2373 (Novavax) | Live | MNT_50_ | Not reported | Available | Not reported | Available |
| Richmond, P, 2021, *Lancet* [41] | SCB-2019 (Clover) | Live | MNT_50_ | 1:20 | Available | $\geq$ 4-fold rise | Available |
| Meng, F, 2021,  *Signal Transduct Target Ther* [36] | Sf9 cells  (West China Hospital) | Live,  pseudo | Not reported | Not reported | Available | $\geq$ 4-fold rise | Not available |
| Shu, Y, 2021, *Chin Med J* [45] | V-01  (Livzon Mabpharm) | Live | MNT_50_ | 1:10 | Available | Seronegative to seropositive, or  $\geq$ 4-fold rise for baseline seropositive | Available |
| Zhang, J, 2021,  *Emerg Microbes Infect* [59] | V-01  (Livzon Mabpharm) | Live | MNT_50_ | 1:10 | Available | Seronegative to seropositive, or  $\geq$ 4-fold rise for baseline seropositive | Not available |
| Yang, S, 2021, *Lancet Infect Dis* [58] | ZF2001 (Anhui Zhifei) | Live | MNT_50_ | 1:4 | Available | Seronegative to seropositive | Available |
| **RNA (n = 9, 24%)** | | | | | | | |
| Li, J, 2021, *Nat Med* [32] | BNT162b1 (Pfizer/BioNTech) | Live | MNT_50_ | 1:10 | Available | $\geq$ 4-fold rise | Available |
| Mulligan, M, 2020, *Nature* [38] | BNT162b1 (Pfizer/BioNTech) | Pseudo | VNT_50_ | Not reported | Not available | -- | Available |
| Sahin, U, 2020, *Nature* [43] | BNT162b1 (Pfizer/BioNTech) | Pseudo | VNT_50_ | Not reported | Not available | -- | Available |
| Walsh, E, 2020, *N Engl J Med* [50] | BNT162b1, BNT162b2 (Pfizer/BioNTech) | Live | MNT_50_ | 1:10 | Not available | -- | Available |
| Walter, E, 2021, *N Engl J Med* [51] | BNT162b2 (Pfizer/BioNTech) | Live | MNT_50_ | Not reported | Not available | -- | Not available |
| Kremsner, P, 2021,  *Wien Klin Wochenschr* [31] | CVnCoV (Curevac) | Live | MNT_50_ | 1:10 | Available | $\geq$ 4-fold rise | Available |
| Anderson, E, 2020, *N Engl J Med* [15] | mRNA-1273 (Moderna) | Pseudo | sVNA IC_50_ | 1:20 | Available | $\geq$ 4-fold rise | Available |
| Chu, L, 2021, *Vaccine* [19] | mRNA-1273 (Moderna) | Live | MNT_50_ | Not reported | Available | Seronegative to seropositive, or  $\geq$ 4-fold rise for baseline seropositive | Available |
| Jackson, L, 2020, *N Engl J Med* [29] | mRNA-1273 (Moderna) | Live,  pseudo | PRNT_80_,  sVNA IC_50_ | 1:8  1:20 | Not available | -- | Available |
| **Non-replicating viral vector (n = 5, 13%)** | | | | | | | |
| Sadoff, J, 2021, *N Engl J Med* [42] | Ad26.COV2.S (Janssen) | Live | MNT_50_ | Not reported | Available | Seronegative to seropositive, or  $\geq$ 4-fold rise for baseline seropositive | Available |
| Wu, S, 2021, *Lancet Infect Dis* [53] | Ad5-nCoV (AMMS) | Live | PRNT_50_ | 1:8 | Available | $\geq$ 4-fold rise | Not available |
| Zhu, F, 2020 (395: 1845-54), *Lancet* [63] | Ad5-nCoV (CanSino) | Live,  pseudo | Not reported,  sVNA IC_50_ | Not reported | Available | $\geq$ 4-fold rise | Not available |
| Zhu, F, 2020 (396: 479-88), *Lancet* [62] | Ad5-nCoV (CanSino) | Live,  pseudo | Not reported,  sVNA IC_50_ | Not reported | Available | $\geq$ 4-fold rise | Not available |
| Zhu, F, 2021, *Clin Infect Dis* [61] | Ad5-nCoV (CanSino) | Pseudo | sVNA IC_50_ | Not reported | Available | $\geq$ 4-fold rise | Not available |
| **DNA (n = 2, 5%)** | | | | | | | |
| Tebas, 2021, *EClinicalMedicine* [48] | INO-4800 (Inovio) | Live | PRNT_50_ | Not reported | Available | Seronegative to seropositive, or  $\geq$ 4-fold rise for baseline seropositive^*^ | Not available |
| Momin, T, 2021, *EClinicalMedicine* [37] | ZyCoV-D  (Zydus Cadila) | Live | PRNT_50_ | Not reported | Available | Seronegative to seropositive, or  $\geq$ 4-fold rise for baseline seropositive | Available |
| **Virus-like particle (n = 1, 3%)** |  |  |  |  |  | |  |
| Ward, B, 2021, *Nat Med* [52] | Plant-based VLP (Medicago) | Live,  pseudo | PRNT_50_,  sVNA IC_50_ | 1:10  1:10 | Available | Not reported | Available |

## Table S4. Summary of 17 studies that are included for cell-mediated response analysis by vaccine type.

| **Reference** | **Vaccine**  **(Manufacturers)** | **Days since full vaccination** | **Dose concentration** | | **Assay**  **(Target)** | **T-cell type** | **Cytokines** | **Baseline available** | **Th1-biased** |
| --- | --- | --- | --- | --- | --- | --- | --- | --- | --- |
|  |  |  | **Standard** | **Examined** |  |  |  |  |  |
| **Inactivated virus (n = 3)** |  |  |  |  |  |  |  |  |  |
| Ella, R, 2021 (21:637-46),  *Lancet Infect Dis [22]* | BBV152  (Covaxin) | 14 | 6 $\mu g$  Algel-IMDG | 3 $\mu g$  Algel-IMDG | ELISpot  (Antigen) | CD4+, CD8+ | IFN-$\gamma$ | Yes | Yes |
| Ella, R, 2021 (21:950-61),  *Lancet Infect Dis* [21] | BBV152  (Covaxin) | 14 | 6 $\mu g$  Algel-IMDG | 3 $\mu g$  Algel-IMDG | Luminex multiplex  (Antigen) | CD4+ | IFN-$\gamma$, TNF-$\alpha$,  IL-2, IL-5, IL-10, IL-13 | No | Yes |
| Pan, H, 2021, *Chin Med J* [40] | KCONVAC  (Minhai BioTech) | 28 | 10 $\mu g$ | 5 $\mu g$ | ELISpot  (Antigen) | NS | IFN-$\gamma$ | Yes | Yes |
| **Protein subunit (n = 4)** |  |  |  |  |  |  |  |  |  |
| Hsieh, S, 2021,  *EClinicalMedicine* [28] | MVCCOV1901  (Medigen) | 28 | 15 $\mu g$ | 5 and 25 $\mu g$ | ELISpot  (Antigen) | NS | IFN-$\gamma$, IL-4 | Yes | Yes |
| Keech, C, 2020, *N Engl J Med* [30] | NVX-CoV2373  (Novavax) | 14 | 5 $\mu g$ | 25 $\mu g$ | ICS  (Antigen) | CD4+ | IFN-$\gamma$, TNF-$\alpha$,  IL-2, IL-5, IL-13 | Yes | Yes |
| Meng, F, 2021,  *Signal Transduct Target Ther* [36] | Sf9 cells  (West China Hospital) | 28 | 20 $\mu g$ | 40 $\mu g$ | ELISpot  (Antigen) | NS | IFN-$\gamma$ | Yes | ND |
| Yang, S, 2021,  *Lancet Infect Dis* [58] | ZF2001  (Anhui Zhifei) | 14 | 50 $\mu g$ | 25 $\mu g$ | ELISpot  (Antigen) | NS | IFN-$\gamma$,  IL-2, IL-4, IL-5 | Yes | No |
| **RNA (n = 4)** |  |  |  |  |  |  |  |  |  |
| Li, J, 2021, *Nat Med* [32] | BNT162b1  (Pfizer/BioNTech) | 7 | 30 $\mu g$ | 10 $\mu g$ | ELISpot  (S1) | NS | IFN-$\gamma$ | Yes | ND |
| Sahin, U, 2020, *Nature* [43] | BNT162b1  (Pfizer/BioNTech) | 7 | 30 $\mu g$ | 1, 10, 50 and 60 $\mu g$ | ELISpot  (RBD) | CD4+, CD8+ | IFN-$\gamma$ | Yes | Yes |
| Anderson, E, 2020, *N Engl J Med* [15] | mRNA-1273  (Moderna) | 14 | 100 $\mu g$ | 25 $\mu g$ | ICS  (Spike) | CD4+, CD8+ | IFN-$\gamma$, TNF-$\alpha$,  IL-2, IL-4, IL-13 | Yes | Yes |
| Jackson, L, 2020, *N Engl J Med* [29] | mRNA-1273  (Moderna) | 14 | 100 $\mu g$ | 25 $\mu g$ | ICS  (Spike) | CD4+, CD8+ | IFN-$\gamma$, TNF-$\alpha$,  IL-2, IL-4, IL-13 | Yes | Yes |
| **Non-replicating viral vector (n = 4)** |  |  |  |  |  |  |  |  |  |
| Sadoff, J, 2021, *N Engl J Med* [42] | Ad26.COV2.S  (Janssen) | 14 | 5$\times$10^10^ VP | 1$\times$10^11^ VP | ICS  (Spike) | CD4+, CD8+ | NS | Yes | Yes |
| Zhu, F, 2020 (395: 1845-54), *Lancet* [63] | Ad5-nCoV  (CanSino) | 28 | 5$\times$10^10^ VP | 1$\times$10^11^ VP  1.5$\times$10^11^ VP | ELISpot  and ICS  (Spike) | CD4+, CD8+ | IFN-$\gamma$, TNF-$\alpha$,  IL-2 | Yes | ND |
| Zhu, F, 2020 (396: 479-88), *Lancet* [62] | Ad5-nCoV  (CanSino) | 28 | 5$\times$10^10^ VP | 1$\times$10^11^ VP | ELISpot  (Spike) | NS | IFN-$\gamma$ | Yes | ND |
| Zhu, F, 2021, *Clin Infect Dis* [61] | Ad5-nCoV  (CanSino) | 56 | 5$\times$10^10^ VP | 1$\times$10^11^ VP | ELISpot  (Spike) | NS | IFN-$\gamma$, IL-2,  IL-4, IL-5, IL-13 | Yes | Yes |
| **DNA (n = 1)** |  |  |  |  |  |  |  |  |  |
| Tebas, 2021, *EClinicalMedicine* [48] | INO-4800  (Inovio) | 14 | 1 $mg$ | 2 $mg$ | ELISpot  (Spike) | NS | IFN-$\gamma$ | Yes | Yes |
| **Virus-like particle (n = 1)** |  |  |  |  |  |  |  |  |  |
| Ward, B, 2021, *Nat Med* [52] | Plant-based VLP  (Medicago) | 21 | 3.75 $\mu g$ | 7.5 and 15 $\mu g$ | ELISpot  (Spike) | NS | IFN-$\gamma$, IL-4 | Yes | No |

*Abbreviation: ELISpot, enzyme-linked immune absorbent spot. ICS, intracellular cytokine staining. RBD, receptor-binding domain. NS, not specified. IFN-$\gamma$, Interferon gamma. TNF-$\alpha$, tumor necrosis factor alpha. IL-2, -4, -5, -10, -13, Interleukin-2, -4, -5, -10, -13. ND, not determined.

## Table S5. Factors associated with neutralizing antibody responses after receiving different fractional doses of vaccinations.

| **Variable** | **Fold of change^*^** |
| --- | --- |
| **Vaccine type** |  |
| Inactivated | Ref |
| RNA | 9.3 (6.8, 12.8) |
| Protein subunit | 4.6 (3.4, 6.2) |
| Non-replicating vector | 7.4 (4.4, 12.4) |
| Virus-like particle | 10.0 (6.1, 16.5) |
| **Assay antigen** |  |
| Live virus | Ref |
| Pseudo virus | 1.3 (1.0, 1.6) |
| **Age group** |  |
| Adult |  |
| Children | 2.5 (1.5, 4.0) |
| Elderly | 0.9 (0.8, 1.2) |
| **Total doses** | 5.2 (3.8, 7.1) |

* The model also adjusted for non-linear effects of days since full vaccination and dose-escalation relationship. Deviance explained by the model was 79%.

## Table S6. Cross-validation of general additive model for dose-response relationship of neutralizing antibody after fractional doses.

| **Subset** | **Sample size of tested subset** | **Pearson correlation** |
| --- | --- | --- |
| **1** | 39 | 0.906 |
| **2** | 42 | 0.853 |
| **3** | 48 | 0.915 |
| **4** | 47 | 0.883 |
| **5** | 50 | 0.841 |
| **6** | 43 | 0.898 |
| **7** | 46 | 0.787 |
| **8** | 46 | 0.883 |
| **9** | 53 | 0.890 |
| **10** | 36 | 0.906 |

## Table S7. Fold of reduction in neutralizing antibodies against variants of concerns.

| **Variant**  **(Pango lineage)** | **Fold of change**  **(95% CI)** | **Reference** |
| --- | --- | --- |
| Alpha (B.1.1.7) | 1.6 (1.5, 1.7) | Cromer et al., 2022[13] |
| Beta (B.1.351) | 8.8 (8.0, 9.7) | Cromer et al., 2022[13] |
| Gamma (P.1) | 3.5 (3.1, 4.0) | Cromer et al., 2022[13] |
| Delta (B.1.617.2) | 3.9 (3.5, 4.4) | Cromer et al., 2022[13] |
| Omicron (B.1.1.529) | 22.8 (21.8, 23.8) | Carreno *et al*. 2021[6] |

## Table S8. Vaccine effectiveness against infections of variants of concern for standard dose.

| **Vaccine** | **Platform** | **Study design^** | **Vaccine effectiveness**  **against infection** | | **Reference** |  |  |
| --- | --- | --- | --- | --- | --- | --- | --- |
|  |  |  | **Symptomatic** | **Severe** |  |  |  |
| **Alpha (B.1.1.7)** |  |  |  |  |  |  |  |
| BNT162b2 | RNA | TND | 89.5 (85.9, 92.3) | 100 (82, 100) | Abu-Raddad, 2021 [14] |  |  |
| BNT162b2 | RNA | TND | 93.7 (91.6, 95.3) | -- | Bernal, 2021 [34] |  |  |
| AZD1222 | Vector | TND | 74.5 (68.4, 79.4) | -- | Bernal, 2021 [34] |  |  |
| NVX-CoV2373 | Subunit | RCT | 86.3 (71.3, 93.5)^*^ | -- | Heath, 2021 [27] |  |  |
| BNT162b2 | RNA | TND | 92 (90, 93) | -- | Sheikh, 2021 [44] |  |  |
| AZD1222 | Vector | TND | 73 (66, 78) | -- | Sheikh, 2021 [44] |  |  |
| BNT162b2 | RNA | TND | 88 (86, 90) | 96 (94, 97) | Nasreen, 2022 [39] |  |  |
| AZD1222 | Vector | TND | 87 (47, 97) | 92 (41, 99) | Nasreen, 2022 [39] |  |  |
| RNA-1273 | RNA | TND | 92 (87, 95) | 95 (92, 97) | Nasreen, 2022 [39] |  |  |
| RNA-1273 | RNA | TND | 99.2 (95.3, 100) | -- | Chemaitelly, 2021 [18] |  |  |
| **Beta (B.1.351)** |  |  |  |  |  |  |  |
| BNT162b2 | RNA | TND | 75 (70.5, 78.9) | 100 (73, 100) | Abu-Raddad, 2021 [14] |  |  |
| AZD1222 | Vector | RCT | 10.4 (-76.8, 54.8) | -- | Madhi, 2021 [35] |  |  |
| NVX-CoV2373 | Subunit | RCT | 51 (-0.6, 76.2) | -- | Shinde, 2021 [30] |  |  |
| BNT162b2 | RNA | TND | 86 (0, 98) | 92 (39, 99) | Nasreen, 2022 [39] |  |  |
| RNA-1273 | RNA | TND | 96.4 (94.3, 97.9) | -- | Chemaitelly, 2021 [18] |  |  |
| BNT162b2 | RNA | TND | 74.3 (70.3, 77.7) | 92.7 (82, 97) | Tang, 2021 [47] |  |  |
| RNA-1273 | RNA | TND | 80.8 (69, 88.2) | 100 (--,--) | Tang, 2021 [47] |  |  |
| **Gamma (P.1)** |  |  |  |  |  |  |  |
| BNT162b2 | RNA | TND | 90 (76, 96) | 94 (59, 99) | Nasreen, 2022 [39] |  |  |
| **Delta (B.1.617.2)** |  |  |  |  |  |  |  |
| BNT162b2 | RNA | TND | 88 (85.3, 90.1) | -- | Bernal, 2021 [34] |  |  |
| AZD1222 | Vector | TND | 67 (61.3, 71.8) | -- | Bernal, 2021 [34] |  |  |
| BNT162b2 | RNA | TND | 79 (75, 82) | -- | Sheikh, 2021 [44] |  |  |
| AZD1222 | Vector | TND | 60 (53, 66) | -- | Sheikh, 2021 [44] |  |  |
| CoronaVac/CNBG | Inactivated | TND | 59 (16, 81.6)^*^ | 100 (--,--) | Li, 2021 [33] |  |  |
| BNT162b2 | RNA | TND | 92 (89, 94) | 95 (64, 99) | Nasreen, 2022 [39] |  |  |
| AZD1222 | Vector | TND | 88 (68, 96) | 90 (67, 97) | Nasreen, 2022 [39] |  |  |
| RNA-1273 | RNA | TND | 94 (90, 97) | 98 (93, 100) | Nasreen, 2022 [39] |  |  |
| BNT162b2 | RNA | TND | 44.4 (37, 50.9) | -- | Tang, 2021 [47] |  |  |
| RNA-1273 | RNA | TND | 73.9 (65.9, 79.9) | -- | Tang, 2021 [47] |  |  |
| RNA-1273 | RNA | TND | 80.2 (68.2, 87.7) | 84.5 (23, 96.9) | Tseng, 2022 [49] |  |  |
| **Omicron (B.1.1.529)** | |  | | | |  |  |
| RNA-1273 | RNA | TND | 44 (35.1, 51.6) | 99 (93.3, 99.9) | Tseng, 2022 [49] |  |  |
| BNT162b2 | RNA | TND | -- | 70 (62, 76) | Collie, 2022 [20] |  |  |

^ TND: test negative design. RCT: randomized clinical trial.

* Outcomes were mostly symptomatic infections.

## Table S9. Factors associated with risk of experiencing seroconversion of neutralizing antibodies after receiving non-standard and standard doses.

|  | **Lower vs. standard dose (%)** | **Higher vs. standard dose (%)** |
| --- | --- | --- |
| **Vaccine platform** |  |  |
| RNA | Ref. | Ref. |
| Inactivated | -3.1 (-5.7, -0.3) | -6.0 (-31.4, 28.8) |
| Protein subunit | -19.7 (-26.9, -11.8) | -7.4 (-32.5, 26.9) |
| Non-replicating vector | -- | -5.4 (-31.4, 30.4) |
| Virus-like particle | -- | -0.8 (-30.1, 40.8) |
| **Age** |  |  |
| Adult | Ref. | Ref. |
| Children | 3.9 (1.1, 6.8) | -1.8 (-3.9, 0.4) |
| Elderly | 0.6 (-1.7, 3.1) | -1.3 (-5.1, 2.6) |
| **Assay antigen** |  |  |
| Live virus | Ref. | Ref. |
| Pseudo virus | 0.5 (-11.8, 14.5) | -0.2 (-12.2, 13.5) |
| **Dose fraction** | 1.4 (-20.4, 29.3) | 0.1 (-1.6, 1.8) |

Data are shown in Additional file 1: Fig. S3.

# Supplementary Figures

**Identification of studies via databases and registers**

Records identified from*:

PubMed (n = 1,733)

44 duplicate records removed *before screening*

**Identification**

1,553 records excluded:

Non-human study (n = 192)

Non-original research (n = 113)

Not COVID-19 (n = 26)

Not COVID-19 vaccine (n = 88)

People with special conditions (n = 326)

No immunological response (n = 639)

No dose escalation (n = 106)

Vaccination after infections (n = 63)

Records screened

(n = 1,689)

**Screening**

97 records excluded:

Duplicate data (n = 2)

No dose escalation (n = 84)

No immunological response (n = 12)

Records assessed for eligibility

(n = 136)

Studies included in:

Review (n = 38)

Seroconversion analysis (n = 32)

Dose-relationship analysis (n = 24)

T-cell analysis (n = 17)

Safety analysis (n = 37)

**Included**

## Fig. S1. Flowchart of literature search and screening.

Among 32 studies which reported seroconversions, 14 compared seroconversion proportion between fractional and standard dose groups, which were shown in Figure 1. The rest 18 studies compared seroconversion proportions between standard and higher dose group, which results can be found in the shared data.

## Fig. S2. Risk of bias of 39 included studies.

The quality of included studies was assessed using the Cochrane Risk of Bias tool 2.0 for randomized trials [46].

**
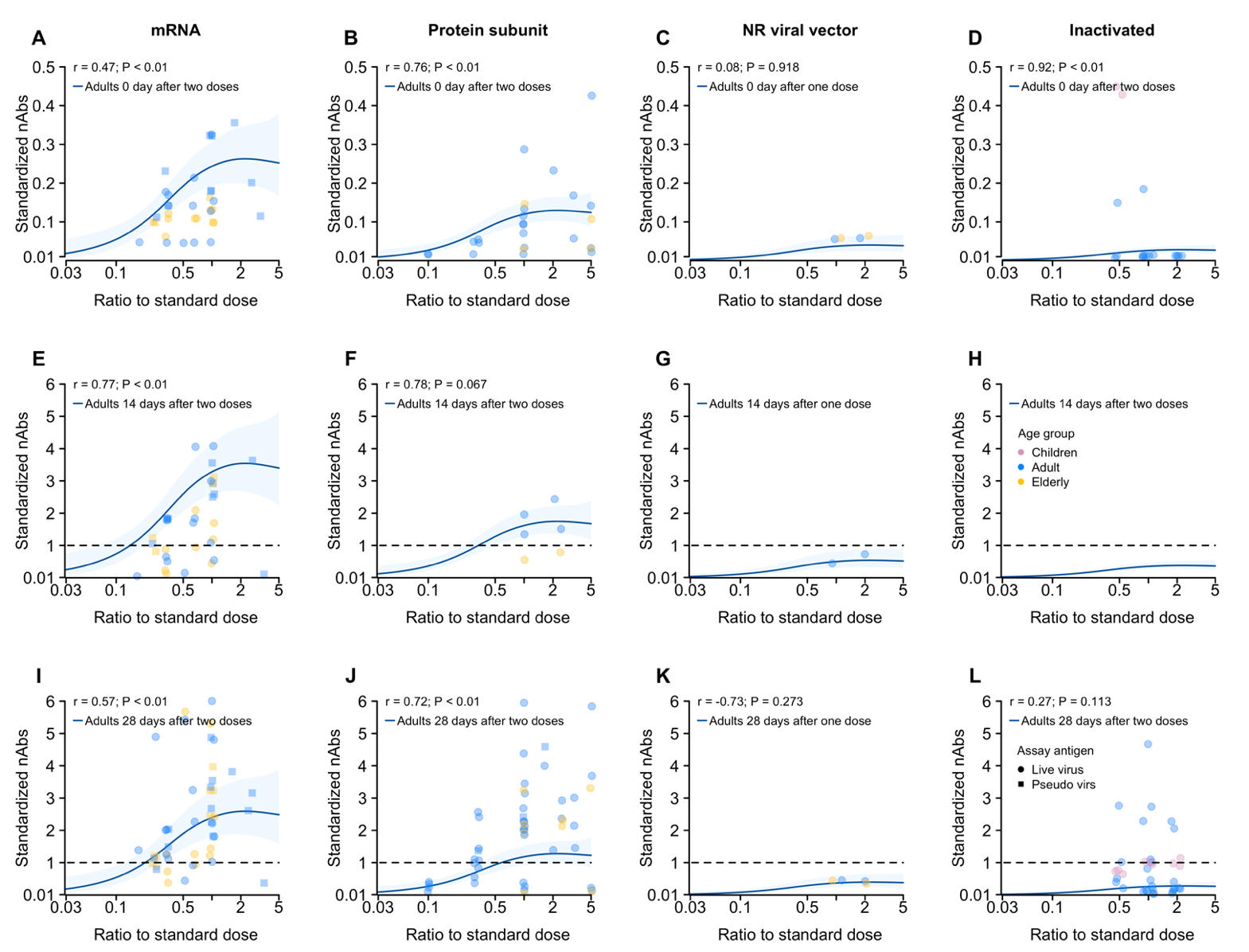
**

## Fig. S3. Model predictions of dose-response relationship of neutralizing antibodies (nAbs) against ancestral strains introduced by COVID-19 vaccines.

nAbs were standardized as the ratio to the convalescent sera. Model estimates were shown in Table S7. Dots indicates the raw data, which were shown in Additional file 1: Figs. S4-S6. We show predictions of nAbs on day 0 (A-D), day 14 (E-H) and day 28 (I-L) since the complete vaccination.

## Fig. S4. Standardized neutralizing antibodies (nAbs) introduced by COVID-19 vaccines on day 0 since the complete vaccination.

nAbs were standardized as the ratio to the convalescent sera. Estimates were classified as groups that received lower (A), the same (B), and higher (C) dose than the standard dose of that vaccine.

## Fig. S5. Standardized neutralizing antibodies (nAbs) introduced by COVID-19 vaccines on day 14 since the complete vaccination.

nAbs were standardized as the ratio to the convalescent sera. Estimates were classified as groups that received lower (A), the same (B), and higher (C) dose than the standard dose of that vaccine.

## Fig. S6. Standardized neutralizing antibodies (nAbs) introduced by COVID-19 vaccines on day 28 or later since the complete vaccination.

nAbs were standardized as the ratio to the convalescent sera. Estimates were classified as groups that received lower (A), the same (B), and higher (C) dose than the standard dose of that vaccine.

## Fig. S7. Associations between time since complete vaccination and the standardized neutralizing antibodies (nAbs) against the ancestral strains elicited by fractioning dose of COVID-19 vaccines.

Predictions on half, standard, and two-fold of the standard vaccine doses are shown. A 2-dose schedule was assumed for RNA, protein subunit and inactivated vaccines, while 1-dose schedule was assumed for non-replicating viral vector, which dose-relationship was driven by a 1-dose schedule vaccine (i.e., Ad26.COV2.S). Complete data that were used to derive these predictions were shown in Additional file 1: Figs. S4-S6. Dashed horizontal line indicates the average level of neutralizing antibodies against the wild-type strain in convalescent sera. (a) RNA. (b) Protein subunit. (c) Non-replicating viral vector. (d) Inactivated.

## Fig. S8. Dose-relationship between dose fractionation and predicted vaccine efficacy against symptomatic infections of variants of concern.


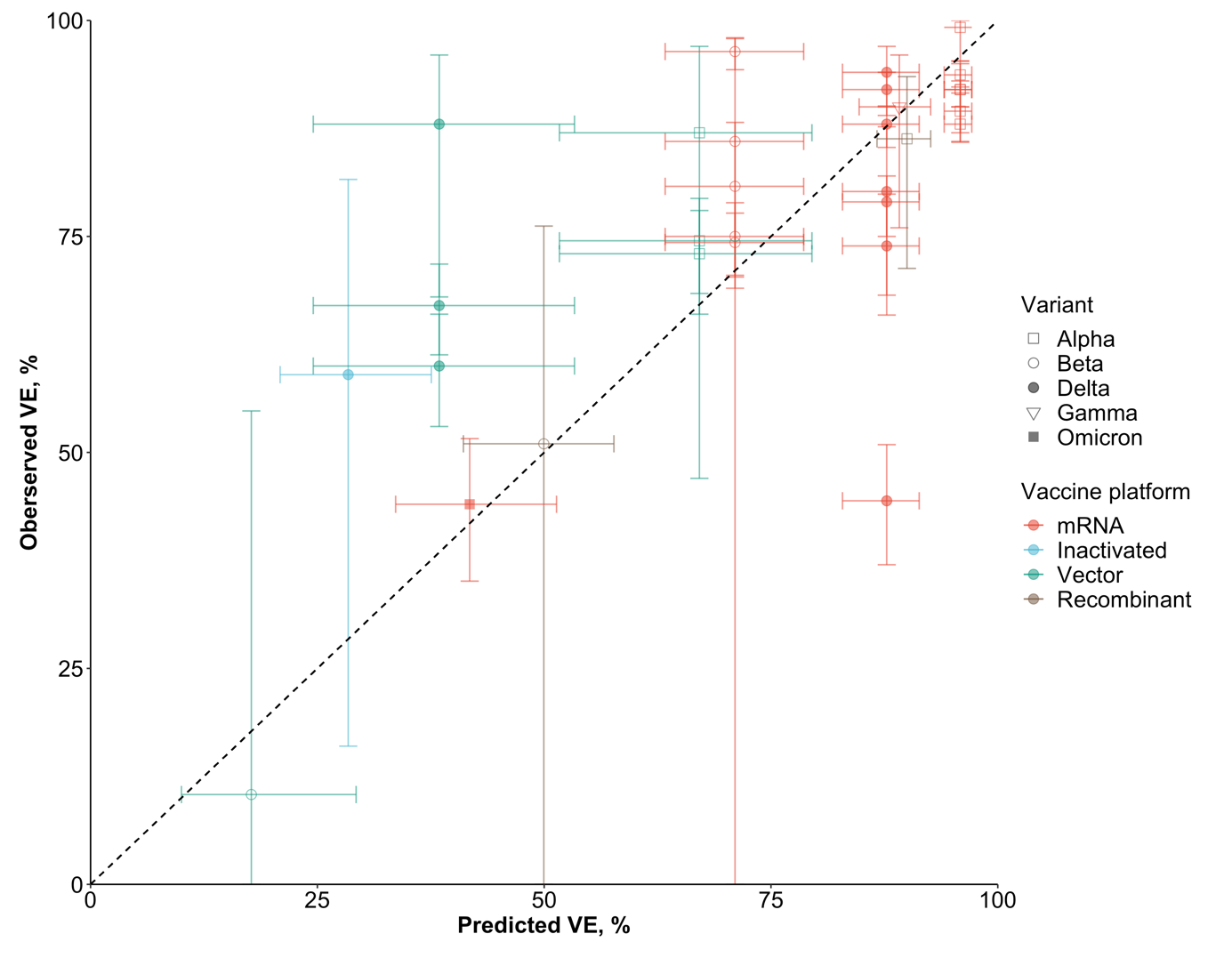


## Fig. S9. Correlation between predicted and observed vaccine efficacy against variants of concern for standard dose of COVID-19 vaccines.

Full model predictions (dose ratio = 1) were shown in Additional file 1: Fig. S8. Observation data were shown in Table S6. Pearson correlation coefficient is 0.705 (p < 0.05).


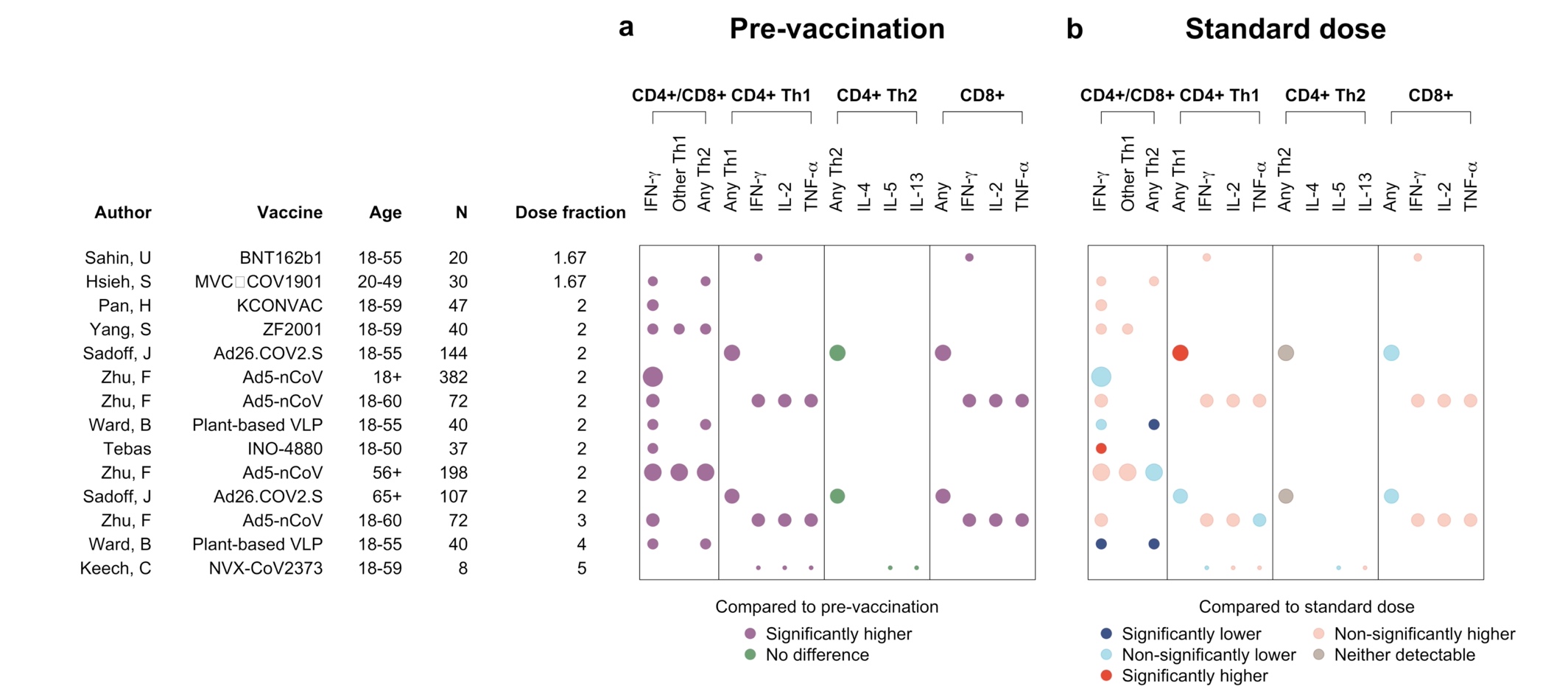


## Fig. S10. Comparison of T-cell responses against the ancestral strains elicited by higher doses of COVID-19 vaccines.

Size of dots represent the total sample sizes of the standard and non-standard dose groups. (a) Compared to pre-vaccination. If the mean and 95% CI of the difference in mean T-cell levels before and after the higher doses were all greater than 0, we determined T-cell responses were significantly higher between the groups. (b) Compared to people who received standard doses. If the mean and 95% CI of the difference in mean T-cell levels between the higher and standard dose groups were all greater or less than 0, we determined T-cell responses were significantly higher or lower than that elicited by the standard dose.

## Fig. S11. Comparison of safety after vaccinated with lower doses (a) and higher doses (b) to standard doses of SARS-CoV-2 vaccines.

Complete data that were used to derive the pooled estimates were shown in Additional file 1: Fig. S12-S16. Dashed horizontal line indicates the average level of adverse events after vaccinated with standard doses of vaccines.

## Fig. S12. Pooled risk ratio (in log scale) of experiencing solicited local adverse events after vaccinated with fractional and standard dose groups.

## Fig. S13. Pooled risk ratio (in log scale) of experiencing solicited systemic adverse events after vaccinated with fractional and standard dose groups.

## Fig. S14. Pooled risk ratio (in log scale) of experiencing any solicited adverse events after vaccinated with fractional and standard dose groups.

## Fig. S15. Pooled risk ratio (in log scale) of experiencing any unsolicited adverse events after vaccinated with fractional and standard dose groups.

## Fig. S16. Pooled risk ratio (in log scale) of experiencing any adverse events after vaccinated with fractional and standard dose groups.
